# Cerebrospinal fluid cytokine-driven immune responses in HIV-negative cryptococcal meningitis

**DOI:** 10.3389/fmed.2026.1800744

**Published:** 2026-04-07

**Authors:** Shi-Ying Zhang, Yu-Ying Pan, Zai-Jie Jiang, Jun-Mei Wang, Bi-Wei Lin, Jian-Chen Hong, Xiang-Ping Yao

**Affiliations:** 1 Department of Neurology, Fujian Institute of Neurology, The First Affiliated Hospital, Fujian Medical University, Fuzhou, China; 2 Department of Gastrointestinal Surgery, The First Affiliated Hospital, Fujian Medical University, Fuzhou, China; 3 Department of Neurology, National Regional Medical Center, Binhai Campus of the First Affiliated Hospital, Fujian Medical University, Fuzhou, China

**Keywords:** cryptococcal meningitis, cerebrospinal fluid, cytokines, immune response, chemokines

## Abstract

Cryptococcal meningitis (CM) is a central nervous system infection caused by *Cryptococcus* species. Although it is readily diagnosable, managing CM remains difficult due to high mortality rates and limited effectiveness of antifungal treatments. Cytokines play a central role in the immunopathogenesis of CM, yet their dynamics in HIV-negative patients remain incompletely characterized. We analyzed CSF cytokine profiles in 28 HIV-negative CM patients and 10 controls with non-CNS inflammatory diseases (NCID) using a 48-plex assay. We found that 39 cytokines increased significantly during infection, while 32 decreased after treatment. Among these, 10 cytokines showed the most pronounced differences. Principal component, correlation and logistic regression analyses were used to characterize these inflammatory cytokines, which exhibited strong positive loading scores on the first principal component and were positively correlated with CSF leukocyte counts. Additionally, monocyte chemotactic protein-3 (MCP-3), monokine induced by interferon gamma (MIG), macrophage inflammatory protein-1 alpha (MIP-1α), and interleukin-18 (IL-18) negatively correlated with the CSF/blood glucose ratio. These observations suggest that specific cytokine patterns are closely linked to central nervous system inflammation and metabolic alterations during CM. Our findings provide comprehensive insight into the immune landscape of HIV-negative CM patients, identify cytokines that may serve as markers of disease activity and treatment response, and could inform future studies exploring targeted immunomodulatory strategies.

## 1 Introduction

Cryptococcal meningitis (CM) is a life-threatening infection of the central nervous system (CNS) caused by *Cryptococcus*, a globally prevalent opportunistic fungal pathogen classified as a “critical threat” on the World Health Organization (WHO) fungal priority pathogen list (1). Over the past decade, diagnostic approaches for CM have advanced from traditional fungal culture and India ink microscopy to cryptococcal antigen (CrAg) detection. More recently, metagenomic next-generation sequencing (mNGS) has emerged as a highly sensitive and specific tool for detecting *Cryptococcus neoformans* (*C. neoformans*) in cerebrospinal fluid (CSF) (2, 3). Despite these diagnostic improvements, CM continues to carry an alarmingly high mortality rate, particularly among HIV-infected individuals, with epidemiological studies reporting fatality rates between 41 and 61% (4). Management remains difficult due to high acute mortality and a limited arsenal of effective antifungal therapies (5).

Although the pathogenesis of CM remains incompletely understood (6), clarifying its underlying mechanisms may enable the development of novel treatment strategies to slow disease progression and improve clinical outcomes. Cytokines are now widely recognized as key mediators of CM immunopathogenesis (7, 8). Previous studies have shown that effective antifungal immunity relies on the host’s ability to detect and control *Cryptococcus* through coordinated interactions between innate immune cells and cell-mediated responses (9). Growing evidence supports the critical role of host immune dynamics in shaping disease manifestation and prognosis (10–12). Jiang et al. reported that in HIV-negative CM, elevated CSF cytokine levels are linked to heightened inflammation and correlate with disease severity (13). Prior studies have implicated dysregulated chemokine signaling in the immune pathogenesis of cryptococcosis-associated immune reconstitution inflammatory syndrome (C-IRIS) (14), where CSF cytokine and chemokine signatures can predict both early mortality and immune reconstitution inflammatory syndrome (IRIS) risk in CM patients (8). In immunocompetent hosts, a Th1/Th17-dominant response is associated with successful clearance of *Cryptococcus* (15), while a Th2-skewed immune profile is often linked to hematogenous dissemination and poor clinical outcomes (7, 8). These findings underscore the pivotal role of cytokines in shaping the immune landscape of CM and influencing disease progression and prognosis. However, the cytokine milieu in HIV-negative CM remains insufficiently characterized.

We hypothesized that distinct cytokines in the CSF contribute differentially to disease processes in HIV-uninfected CM patients. To investigate this, we quantified cytokine concentrations in the CSF of 28 CM patients and 10 individuals with non-CNS inflammatory diseases (NCID). We then compared cytokine profiles before and after antifungal treatment and assessed their associations with clinical laboratory parameters. Our goal was to gain insight into the immune landscape of these patients and identify cytokines that could serve as potential markers of disease activity or treatment response, thereby informing future studies on immunopathogenic mechanisms and therapeutic strategies.

## 2 Materials and methods

### 2.1 Study cohort

In this retrospective study, we consecutively enrolled 29 HIV-negative patients diagnosed with CM at the First Affiliated Hospital of Fujian Medical University between August 2021 and February 2024. All CM patients met at least one of the following diagnostic criteria: positive CSF smear for *Cryptococcus*, positive CSF culture, positive mNGS results, or a positive CSF CrAg test using the IMMY Cryptococcal Antigen Lateral Flow Assay (Immuno-Mycologics) (16). Patients were excluded if they had other central nervous system infections, active systemic infections, or acute autoimmune neurological or systemic inflammatory diseases. Patients refused to undergo lumbar puncture or had contraindications to lumbar puncture were also excluded. Clinical background information was systematically recorded for all participants, including comorbidities, ongoing medications, and prior medical history. Common chronic conditions such as hypertension, diabetes mellitus, and chronic kidney disease were documented. Some patients had stable autoimmune diseases or were receiving long-term immunosuppressive therapy; these patients were included only if their condition was clinically stable and without evidence of acute CNS infection at enrollment. Additionally, we assigned 10 patients with CSF leukocyte counts <5 × 10^6^/L and no evidence of CNS infection to the NCID control group. All participants tested seronegative for HIV. Clinical and laboratory data were collected at baseline, at the time of successful treatment response, and during scheduled follow-up visits at approximately 4, 8, and 12 weeks after treatment initiation. These longitudinal data were recorded to enable dynamic analysis of disease progression and treatment response. Successful treatment response was defined as survival within the observation period, improvement or resolution of attributable symptoms and signs, normalization or improvement of CSF chemistry and cell count, and negative CSF culture. Patients who did not have a lumbar puncture at that particular time point were evaluated based only on clinical symptoms and signs (17).

### 2.2 Specimen handling and routine CSF testing

We performed lumbar punctures using atraumatic needles and collected 10–20 mL of CSF per patient. Within 1 hour of collection, we centrifuged the samples at 500 × *g* for 10 min at 4 °C. Then, we aliquoted the supernatant into 500 μL portions using polypropylene tubes and stored the samples at −80 °C until analysis. CSF evaluation included routine, biochemical, cytological, and oligoclonal band testing, along with microbiological assessment for *Cryptococcus* by fungal culture, smear, and India ink staining. All laboratory procedures were conducted at the Laboratory Medicine Center of the First Affiliated Hospital of Fujian Medical University.

Except for one CM patient whose CSF volume was insufficient, we performed cytokine profiling on CSF samples from the remaining patients. Notably, we collected paired CSF samples before and after antifungal treatment from 15 of the 28 CM patients and analyzed changes in cytokine profiles.

### 2.3 Measurement of cytokines

Luminex liquid suspension chip assays were conducted by Wayen Biotechnologies (Shanghai, China). All CSF samples were analyzed at first thaw. We used an undiluted 50 μL aliquot of CSF supernatant for testing. Cytokine concentrations were measured using the Bio-Plex Pro Human Cytokine 48-plex Panel (Bio-Rad), including: basic fibroblast growth factor (Basic FGF), cutaneous T cell-attracting chemokine (CTACK), eosinophil chemotactic protein (Eotaxin), granulocyte colony-stimulating factor (G-CSF), granulocyte-macrophage colony-stimulating factor (GM-CSF), growth-related oncogene-alpha (GRO-*α*), hepatocyte growth factor (HGF), interferon (IFN)-α2, IFN-γ, interleukin (IL)-10, IL-12 (p40), IL-12 (p70), IL-13, IL-15, IL-16, IL-17, IL-18, IL-1α, IL-1β, IL-1rα, IL-2, IL-2rα, IL-3, IL-4, IL-5, IL-6, IL-7, IL-8, IL-9, interferon-inducible protein-10 (IP-10), Leukemia inhibitory factor (LIF), macrophage-colony stimulating factor (M-CSF), monocyte chemotactic protein (MCP)-1, MCP-3, macrophage migration inhibitory factor (MIF), monokine induced by IFN-γ (MIG), macrophage inflammatory protein (MIP)-1α, MIP-1β, platelet-derived growth factor-BB (PDGF-BB), regulated on activation, normal T cell expressed and secreted (RANTES), stem cell factor (SCF), stem cell growth factor-beta (SCGF-β), stromal-derived factor 1alpha (SDF-1α), tumor necrosis factor (TNF)-α, TNF-β, TNF-related apoptosis-inducing ligand (TRAIL), vascular endothelial growth factor (VEGF) and beta-nerve growth factor (β-NGF) (18), which is a bead-based immunoassay that enables simultaneous quantification of 48 immune mediators from a single sample. (19). Following assay completion, we analyzed the samples and standards using the Luminex 200 system (Luminex Corporation, Austin, TX, USA). The system automatically processed the fluorescent signal data and generated output files in Excel format. All cytokine concentrations were reported in picograms per milliliter (pg/mL). For statistical analysis, we applied standardized handling for values outside the assay’s detection range. Specifically, concentrations below the lower limit of detection were imputed as 0.5× the lowest value of the standard curve, while concentrations exceeding the upper limit were assigned a value of 2× the highest point on the standard curve (20).

### 2.4 Statistical analysis

Patients were stratified based on baseline intracranial pressure (ICP) and infection persistence. Elevated ICP was defined as CSF opening pressure >250 mm H_2_O, and normal ICP as ≤250 mm H_2_O, measured at the diagnostic lumbar puncture prior to antifungal therapy (21, 22). Clinical outcomes were classified as survived and died, determined by follow-up records.

We presented continuous variables as medians with interquartile ranges (IQRs) and summarized categorical variables as counts and percentages. For continuous data following a normal distribution, we applied Student’s *t*-test; for non-normally distributed data, we used the Mann–Whitney U test. Cytokine concentrations (pg/mL) were log-transformed prior to statistical analysis. We generated heatmaps of cytokine correlations using the corrplot package (version 1.0.12) in R, calculating correlation coefficients via the Pearson method. To reduce dimensionality and minimize multiple comparisons, we performed principal component analysis (PCA) on CSF cytokine concentrations from untreated CM patients. PCA extracted linear, orthogonal principal components (PCs) that capture the variance within the dataset. We assessed associations between PCs and CSF parameters, including protein concentration, leukocyte count, glucose levels, and CSF/blood glucose ratio, using Spearman or Pearson correlation analyses and logistic regression, as appropriate. We applied identical methods to evaluate correlations between individual CSF cytokine levels and CSF parameters. We defined statistical significance as a two-tailed *p*-value less than 0.05. FDR-corrected *p*-values are reported and FDR-*p* < 0.05 was considered significant. FDR adjustment was done using the Benjamini and Hochberg approach (23). All analyses were conducted using GraphPad Prism version 10.0 (GraphPad Software Inc., San Diego, CA, USA) and R version 4.2.1.

## 3 Results

### 3.1 Clinical characteristics of enrolled patients

We retrospectively analyzed data from 39 participants, including 28 patients diagnosed with CM, 10 with NCID, and one CM patient whose CSF sample volume was insufficient for cytokine analysis (Supplementary Figure S1). A comprehensive summary of the clinical characteristics of all enrolled patients is provided in Table 1 and Supplementary Table S1. All CM cases were confirmed by the detection of *Cryptococcus neoformans* in CSF samples (*n* = 28). The NCID group included the following diagnoses: vascular parkinsonism (1 case), Alzheimer’s disease (2 cases), narcolepsy with cataplexy (1 case), epileptic seizures (1 case), transient ischemic attack (1 case), low intracranial pressure (1 case), extraocular muscle paralysis (1 case), cerebral infarction (1 case), and Parkinson’s disease (1 case). The median age in the CM group was 60.5 years, with 57.1% (16/28) male patients. In the NCID group, the median age was 58 years, and 20.0% (2/10) were male (Table 2). Age and sex distributions did not differ significantly between the two groups. The most common presenting symptoms in CM patients were fever (60.7%), headache (92.9%), and vomiting (46.4%). Significant differences in CSF parameters were observed between the CM and NCID groups, including intracranial pressure (190 vs. 103 mm H_2_O, *p* = 0.0039), CSF leukocyte count (112.5 × 10^6^/L vs. 1 × 10^6^/L, *p* < 0.0001), CSF glucose level (1.8 vs. 3.4 mmol/L, *p* = 0.0011), CSF protein concentration (900 mg/L vs. 330 mg/L, *p* < 0.0001), and CSF/blood glucose ratio (%) (30.75 vs. 66.00, *p* < 0.0001) (Table 2).

**Table 1:** Clinical characteristics, diagnostic test results, and clinical outcomes of the enrolled patients.

No.	Sex	Age (years)	Comorbidities	SD	Main complaint	IIS	Cul	CrAg	mNGS	Responses to therapy	Final diagnosis	Clinical outcome
1	M	66	Autoimmune disease with immunosuppressive therapy	10	Fever, headache	+	+	−	*C.n*	+	CM	Survived
2	M	71	Malignancy	10	Headache	+	+	−	*C.n*	+	CM	Survived
3	M	47	None	14	Recurrent fever with headache	+	+	−	−	+	CM	Survived
4	F	36	Immunosuppressive therapy	7	Recurrent headache	+	+	+	*C.n*	+	CM	Survived
5	F	67	Hypertension	10	Fever with headache	+	−	−	*C.n*	+	CM	Survived
6	M	62	None	60	Headache, unconsciousness	−	−	+	−	+	CM	Survived
7	F	54	Autoimmune disease	4	Slurred speech	+	+	+	−	+	CM	Survived
8	M	40	Hypertension	60	Headache	+	+	+	*C.n*	+	CM	Survived
9	M	73	None	90	Fever and headache	+	+	+	*C.n*	+	CM	Survived
10	F	59	None	60	Fever and headache	+	+	+	*C.n*	+	CM	Survived
11	M	39	Autoimmune disease with immunosuppressive therapy	14	Headache	+	−	+	*C.n*	+	CM	Survived
12	F	49	Autoimmune disease with immunosuppressive therapy	60	Recurrent headache and vomiting	−	+	+	*C.n*	+	CM	Survived
13	F	72	Other opportunistic infections	10	Headache and fatigue	+	+	+	−	+	CM	Survived
14	M	61	Hypertension	14	Headache and fever	−	−	+	*C.n*	+	CM	Survived
15	M	64	None	60	Headache	−	−	+	−	+	CM	Survived
16	M	65	Autoimmune disease with immunosuppressive therapy	30	Recurrent fever	+	+	+	*C.n*	+	CM	Survived
17	M	27	None	10	Recurrent headache	+	+	+	*C.n*	+	CM	Survived
18	F	59	Hypertension, diabetes and autoimmune disease with immunosuppressive therapy	30	Recurrent headache and vomiting	+	+	+	−	+	CM	Died
19	F	50	None	7	Recurrent headache	+	+	+	*C.n*	+	CM	Survived
20	M	72	Hypertension	20	Headache	+	+	−	−	+	CM	Survived
21	F	40	None	2	Abnormal behavior	+	+	+	−	+	CM	Survived
22	F	48	Hypertension	21	Headache	+	+	+	*C.n*	+	CM	Died
23	F	70	Diabetes, other opportunistic infections and autoimmune disease with immunosuppressive therapy	60	Cough and sputum	+	+	+	−	+	CM	Died
24	M	60	None	90	Recurrent headache	+	+	+	−	+	CM	Survived
25	M	61	Autoimmune disease with immunosuppressive therapy	60	Headache with fever	+	+	+	−	+	CM	Survived
26	F	71	Hypertension	30	Recurrent headache and vomiting	+	+	+	*C.n*	+	CM	Survived
27	M	55	Hypertension and diabetes	20	Headache, dizziness and unstable walking	+	+	+	−	+	CM	Survived
28	M	61	Other opportunistic infections	60	Weakness of limbs	+	+	+	*C.n*	+	CM	Died
29	M	76	NA	30	Difficulty walking	−	−	−	−	NA	Vascular parkinsonism	Survived
30	F	58	NA	730	Memory loss	−	−	−	−	NA	Alzheimer’s disease	Survived
31	M	70	NA	2,920	Repeated episodes of limb weakness	−	−	−	−	NA	Narcolepsy and cataplexy	Survived
32	F	32	NA	30	Transient loss of consciousness	−	−	−	−	NA	Epileptic seizures	Survived
33	F	54	NA	30	Paroxysmal left limb weakness	−	−	−	−	NA	Transient ischemic attack	Survived
34	F	39	NA	7	Recurrent headache	−	−	−	−	NA	Low intracranial pressure	Survived
35	F	65	NA	10	Headache, dizziness with diplopia	−	−	−	−	NA	Extraocular muscle paralysis	Survived
36	F	60	NA	1,460	Memory loss	−	−	−	−	NA	Alzheimer’s disease	Survived
37	F	46	NA	30	Right limb weakness	−	−	−	−	NA	Cerebral infarction	Survived
38	F	58	NA	370	Slow walking	−	−	−	−	NA	Parkinson’s disease	Survived

No., case number; M, male; F, female; SD, symptom duration before admission (days); IIS, India ink stain; Cul, culture; CrAg, cryptococcal antigen; mNGS, metagenomic next-generation sequence; NA, not available; −, negative; +, positive; *C.n*, *Cryptococcus neoformans*; CM, cryptococcal meningitis.

**Table 2:** Clinical characteristics and CSF laboratory parameters in CM and NCID patients.

Variable	CM	NCID	*p*-value*
*n*	28	10	—
Age, years, median (range)	60.5 (27–73)	58.0 (32–76)	0.7827
Gender, male/female (%)	16/12 (57.1)	2/8 (20.0)	0.0673
Symptoms			
Fever, *n* (%)	17 (60.7)	—	—
Headache, *n* (%)	26 (92.9)	—	—
Vomiting, *n* (%)	13 (46.4)	—	—
Altered mental status, *n* (%)	7 (25.0)	—	—
CSF findings, median (range)			
Intracranial pressure, mm H_2_O	190 (30–330)	103 (70–162)	0.0039
Leukocyte count, ×10^6^/L	112.5 (26–364)	1 (0–3)	<0.0001
Protein concentration, mg/L	900 (410–3,010)	330 (190–950)	<0.0001
Glucose level mmol/L	1.8 (2.7–5.6)	3.4 (2.7–5.6)	0.0011
CSF/blood glucose ratio (%)	30.75 (0.03–87.80)	66.00 (49.00–90.00)	<0.0001

CM, cryptococcal meningitis; NCID, non-CNS inflammatory diseases; CSF, cerebrospinal fluid. *Were compared between CM and NCID groups using Mann–Whitney U test.

Table 3 summarizes the changes in clinical manifestations and CSF parameters following antifungal therapy. All 15 CM patients demonstrated a positive clinical response to treatment. Post-treatment analysis revealed significant improvements in CSF leukocyte count (*p* < 0.0001), glucose level (*p* = 0.0011), protein concentration (*p* < 0.0001), and CSF/blood glucose ratio (%) (*p* < 0.0001) compared to pre-treatment values. Supplementary Table S2 shows the details of the CSF profile change after antifungal therapy. While most patients showed clinical improvement during hospitalization, 4 CM patients ultimately died (Table 1).

**Table 3:** Main symptoms and changes in CSF laboratory parameters in UCM and TCM patients (*n* = 15).

Variable	UCM	TCM	*p*-value*
Symptoms			
Fever, *n* (%)	10 (66.7)	0 (0)	—
Headache, *n* (%)	14 (93.3)	0 (0)	—
Vomiting, *n* (%)	6 (40.0)	0 (0)	—
Altered mental status, *n* (%)	4 (26.7)	0 (0)	—
CSF findings, median (range)			
Intracranial pressure, mm H_2_O	169 (80–320)	175 (95–330)	0.0039
Leukocyte count, ×10^6^/L	110 (26–274)	15 (3–48)	<0.0001
Protein concentration, mg/L	790 (410–2,980)	410 (100–1,630)	<0.0001
Glucose level, mmol/L	1.8 (0.5–4.0)	3.0 (1.8–3.7)	0.0011
CSF/blood glucose ratio (%)	33.40 (8.78–54.40)	36.91 (26.39–90.31)	<0.0001

UCM, untreated cryptococcal meningitis; TCM, treated cryptococcal meningitis; CSF, cerebrospinal fluid. *Were compared between UCM and TCM groups using Mann–Whitney U test.

### 3.2 Cytokine profiles in the CSF of enrolled patients

**Figure 1:**
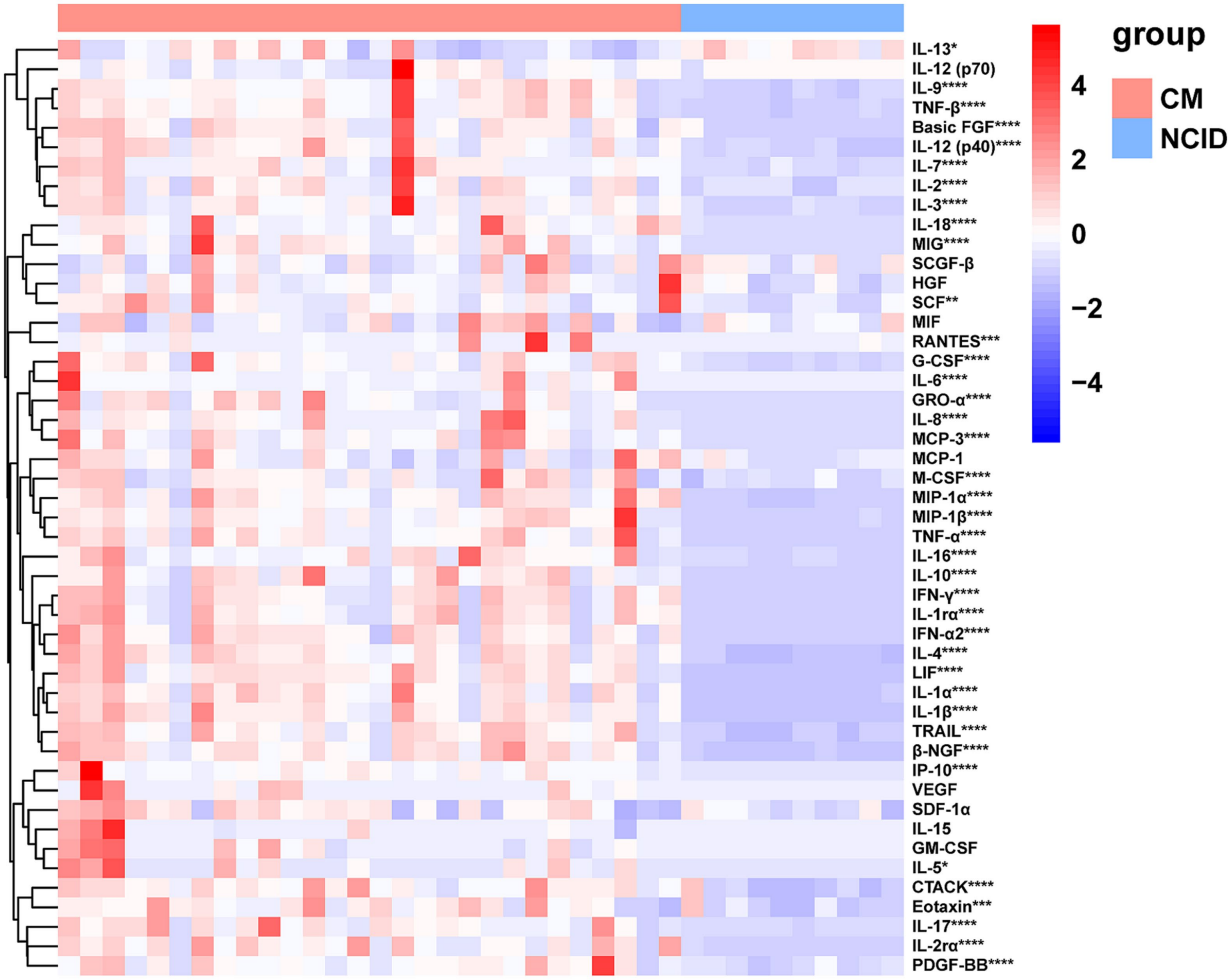
Heat map of cytokine profiles in the CSF of enrolled patients. CM: *n* = 28, NCID: *n* = 10. The levels of cytokines in CSF were measured by the Bio-Plex Pro Human Cytokine Screening 48-plex Panel. Significant cytokines between CM and NCID (*p* < 0.05) were indicated with an asterisk. *p < 0.05, **p < 0.01, ***p < 0.001, ****p < 0.0001. CSF, cerebrospinal fluid; CM, cryptococcal meningitis; NCID, non-CNS inflammatory diseases; FGF, Fibroblast Growth Factor; CTACK, cutaneous T cell-attracting chemokine; Eotaxin, eosinophil chemotactic protein; G-CSF, granulocyte colony-stimulating factor; GRO, rowth-related oncogene; IFN, interferon; IL, interleukin; IP, IFN-*γ* inducible protein; LIF, leukemia inhibitory factor; M-CSF, macrophage-colony stimulating factor; MCP, monocyte chemotactic protein; MIG, monokine induced by IFN-γ; MIP, macrophage inflammatory protein; PDGF, platelet-derived growth factor; RANTES, regulated upon activation normal T cell expressed and secreted factor; SCF, stem cell factor; TNF, tumor necrosis factor; TRAIL, TNF-related apoptosis-inducing ligand; NGF, nerve growth factor.

**Figure 2:**
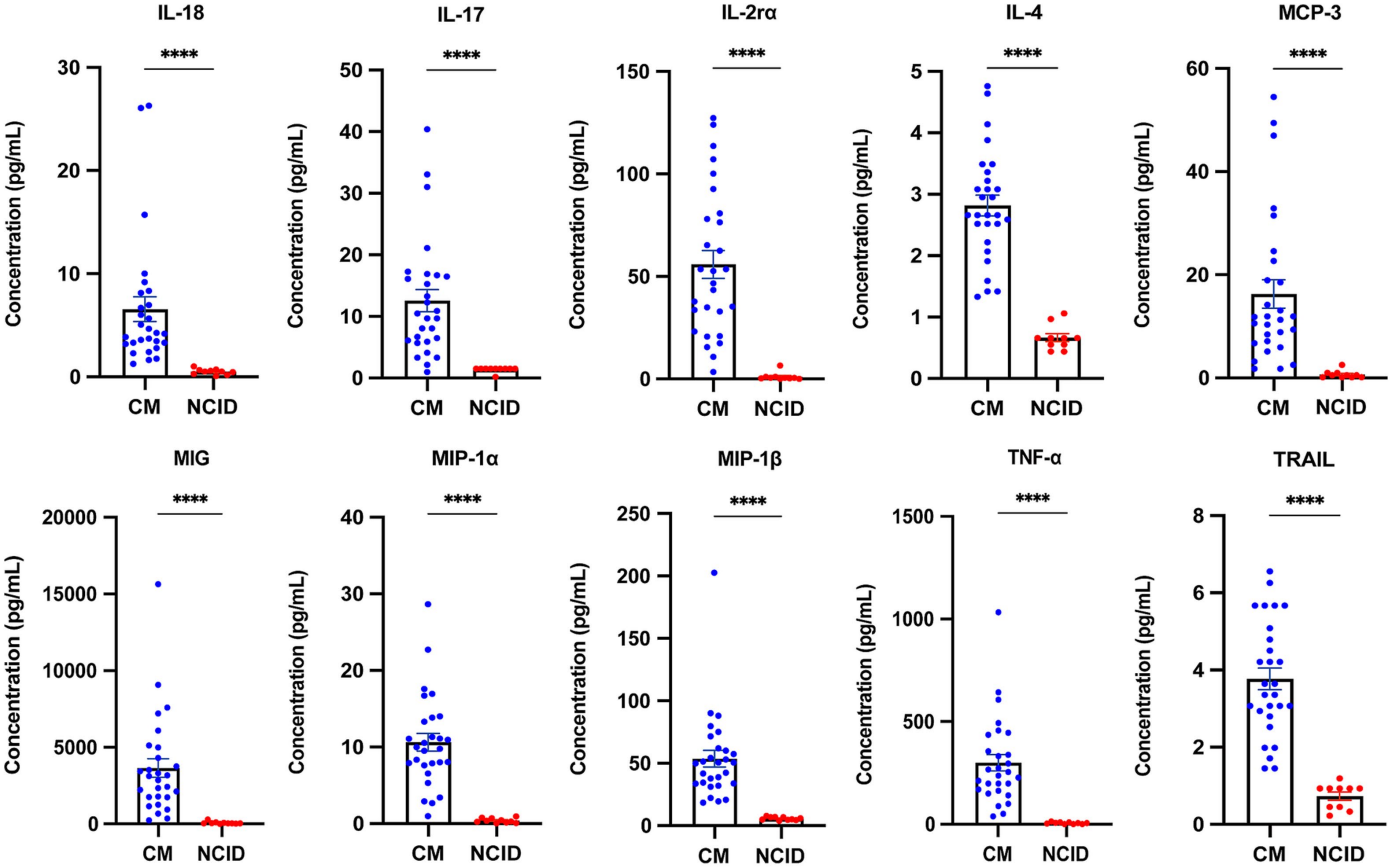
Comparison of CSF cytokine concentrations between the CM and NCID. The data in the graphs were reported as mean ± SEM for each sample group. Comparisons between the two groups were performed using either the Student’s t-test or Mann–Whitney *U* test, as appropriate (****p < 0.0001). CSF, cerebrospinal fluid; CM, cryptococcal meningitis; NCID, non-CNS inflammatory diseases; IL, interleukin; MCP, monocyte chemotactic protein; MIG, monokine induced by interferon gamma; MIP, macrophage inflammatory protein; TNF, tumor necrosis factor; TRAIL, TNF-related apoptosis-inducing ligand.

Of the 48 cytokines analyzed, 39 showed significantly elevated concentrations in the CM group compared to the NCID group, including Basic FGF, CTACK, Eotaxin, G-CSF, GRO-*α*, IFN-α2, IFN-γ, IL-10, IL-12 (p40), IL-13, IL-16, IL-17, IL-18, IL-1α, IL-1*β*, IL-1rα, IL-2, IL-2rα, IL-3, IL-4, IL-5, IL-6, IL-7, IL-8, IL-9, IP-10, LIF, M-CSF, MCP-3, MIG, MIP-1α, MIP-1β, PDGF-BB, RANTES, SCF, TNF-α, TNF-β, TRAIL, and β-NGF (all *p* < 0.05) (Supplementary Table S3; Figures 1, 2). Based on baseline intracranial pressure (high vs. normal) and clinical outcome (survived vs. died), patients were further stratified to explore potential associations with CSF cytokine levels. Although several cytokines showed differences between groups (*p* < 0.05), none remained significant after FDR correction. These findings reflect a robust inflammatory cytokine milieu in cryptococcal meningitis. Given the small sample size for each subgroup, these stratified analyses should be interpreted as descriptive and exploratory.

### 3.3 Dynamics of cytokines after antifungal treatment

**Figure 3:**
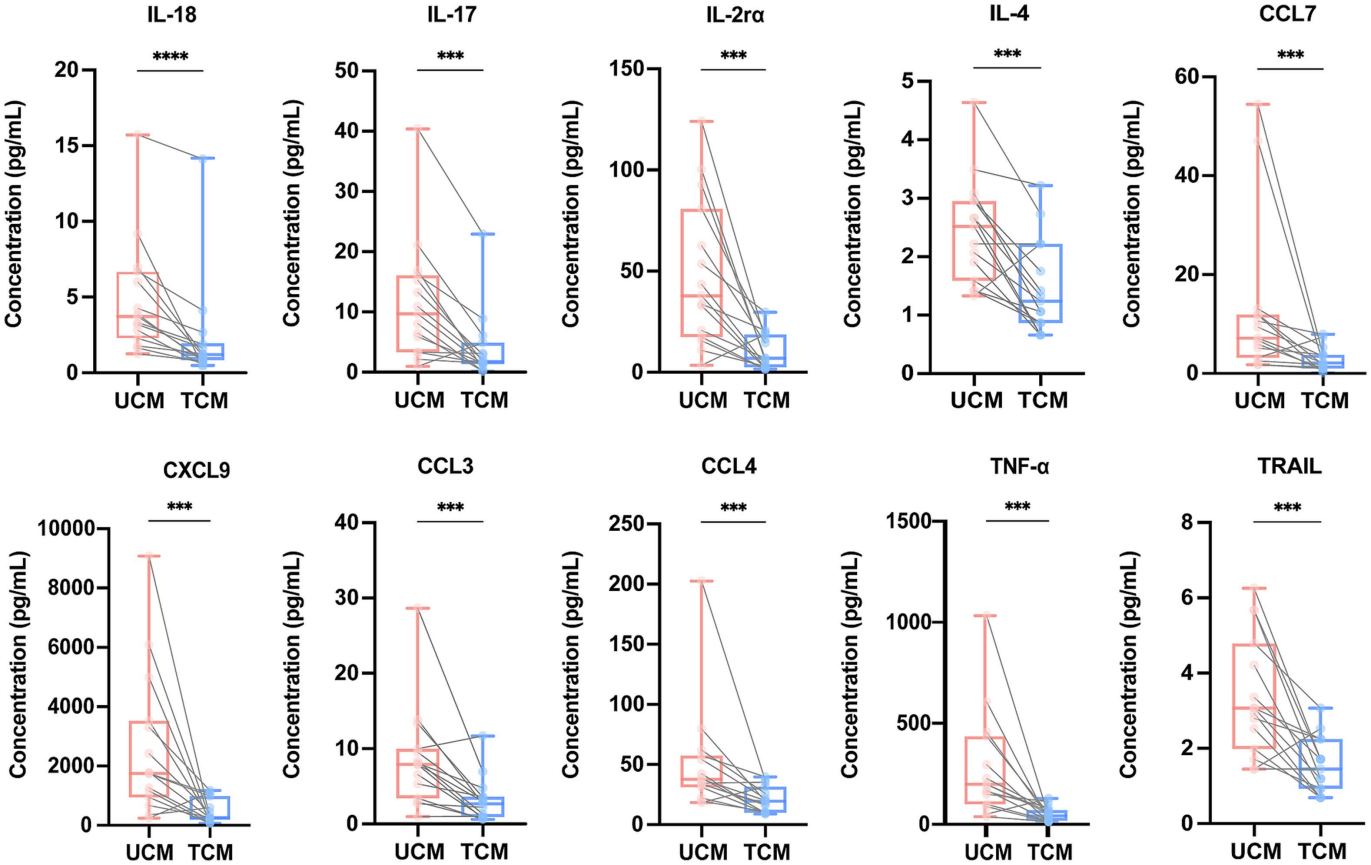
Changes in CSF cytokine concentrations in patients with UCM and TCM. Boxplots show the distribution of cytokine concentrations in the UCM and TCM groups and connect paired samples from the same individual before and after antifungal therapy. Comparisons between paired samples were performed using either the paired t-test or Wilcoxon signed-rank test, as appropriate. ***p < 0.001, ****p < 0.0001. CSF, cerebrospinal fluid; UCM, untreated cryptococcal meningitis; TCM, treated cryptococcal meningitis; IL, interleukin; MCP, monocyte chemotactic protein; MIG, monokine induced by interferon gamma; MIP, macrophage inflammatory protein; TNF, tumor necrosis factor; TRAIL, TNF-related apoptosis-inducing ligand.

We evaluated cytokine levels in CSF samples from 15 CM patients after antifungal therapy. In parallel with clinical improvement (Table 3), cytokine concentrations were generally lower in the treated CM (TCM) group compared to the untreated CM (UCM) group. Of the 39 previously elevated cytokines, 32 demonstrated statistically significant reductions after treatment, including Basic FGF, G-CSF, GRO-*α*, IFN-α2, IFN-*γ*, IL-10, IL-12 (p40), IL-16, IL-17, IL-18, IL-1α, IL-1*β*, IL-1rα, IL-2, IL-2α, IL-3, IL-4, IL-6, IL-7, IL-8, IL-9, IP-10, LIF, MCP-3, MIG, MIP-1α, MIP-1β, SCF, TNF-α, TNF-β, TRAIL, and β-NGF (Supplementary Table S3). Notably, ten cytokines exhibited the most pronounced differences between the TCM and UCM groups: IL-18, IL-17, IL-2rα, IL-4, MCP-3, MIG, MIP-1α, MIP-1β, TNF-α, and TRAIL (Figure 3). These cytokine changes reflect a post-treatment reduction in CSF cytokine concentrations, consistent with improvement in the inflammatory milieu, and may represent potential indicators of treatment response in cryptococcal meningitis.

### 3.4 Identification of cytokines associated with CM

**Figure 4:**
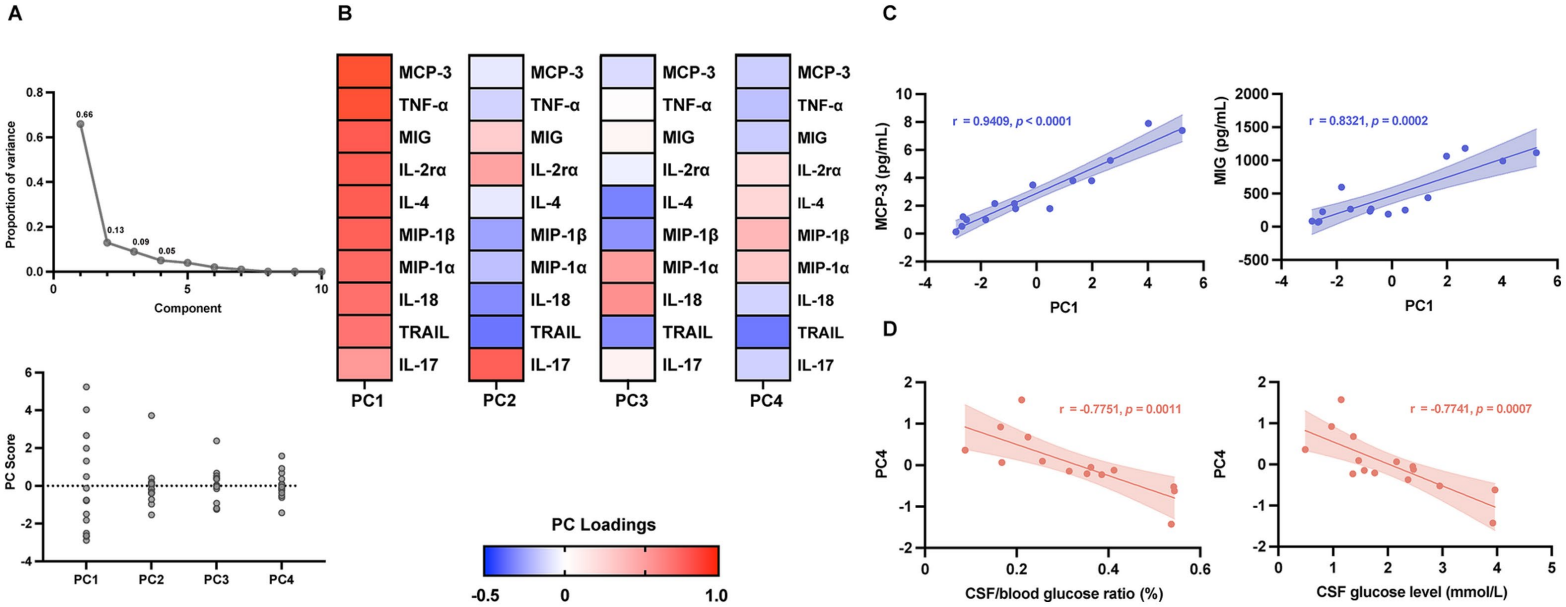
Principal component analysis of CSF cytokine profiles in patients with UCM. **(A)** Proportion of variance explained by each principal component, as well as the variability in principal component scores among the 15 UCM patients included in the study; **(B)** Heat map showing the PC loadings of each CSF cytokine contributing to the first four principal components (PC1–PC4), and color intensity represents the magnitude of the loadings; **(C)** PC1 was positively correlated with the concentrations of MCP-3 and MIG; **(D)** CSF/blood glucose ratio (%) and CSF glucose level (mmol/L) were negatively correlated with PC4. Each dot represents an individual patient sample. Solid lines indicate linear regression fits and shaded areas represent the 95% confidence intervals. Correlation coefficients (*r*) and corresponding *p* values were calculated using Spearman’s correlation analysis. CSF, cerebrospinal fluid; UCM, untreated cryptococcal meningitis; PC, principal component; MCP, monocyte chemotactic protein; TNF, tumor necrosis factor; MIG, monokine induced by interferon gamma; IL, interleukin; MIP, macrophage inflammatory protein; TRAIL, TNF-related apoptosis-inducing ligand.

We conducted PCA to evaluate the variance among co-correlated cytokines in 15 UCM patients and to explore their association with disease characteristics. Four principal components [PC1 (65.81%), PC2 (13.12%), PC3 (8.71%) and PC4 (4.93%)] accounted for 92.70% of the total variance (Figure 4A). Component loadings indicated that PC1 was strongly positively influenced by MCP-3, TNF-*α*, MIG, IL-2rα, IL-4, MIP-1β, MIP-1α, IL-18, and TRAIL, and moderately by IL-17. PC2 was primarily driven by IL-17. PC3 showed moderate positive loadings for MIP-1α and IL-18, while PC4 was positively influenced by MIP-1α and MIP-1β and negatively by TRAIL (Figure 4B). PC1 scores demonstrated strong positive correlations with MCP-3 and MIG levels (Figure 4C). We further assessed associations between PC scores and CSF parameters—specifically protein concentration, leukocyte count, glucose level, and CSF/blood glucose ratio. Notably, PC4 scores were negatively correlated with both CSF glucose (*r* = −0.7741, *p* = 0.0007) and CSF/blood glucose ratio (%) (*r* = −0.7751, *p* = 0.0011) (Figure 4D), suggesting a potential link with aspects of CSF metabolic status.

### 3.5 Correlations between CSF cytokine levels and parameters in CM patients

**Figure 5:**
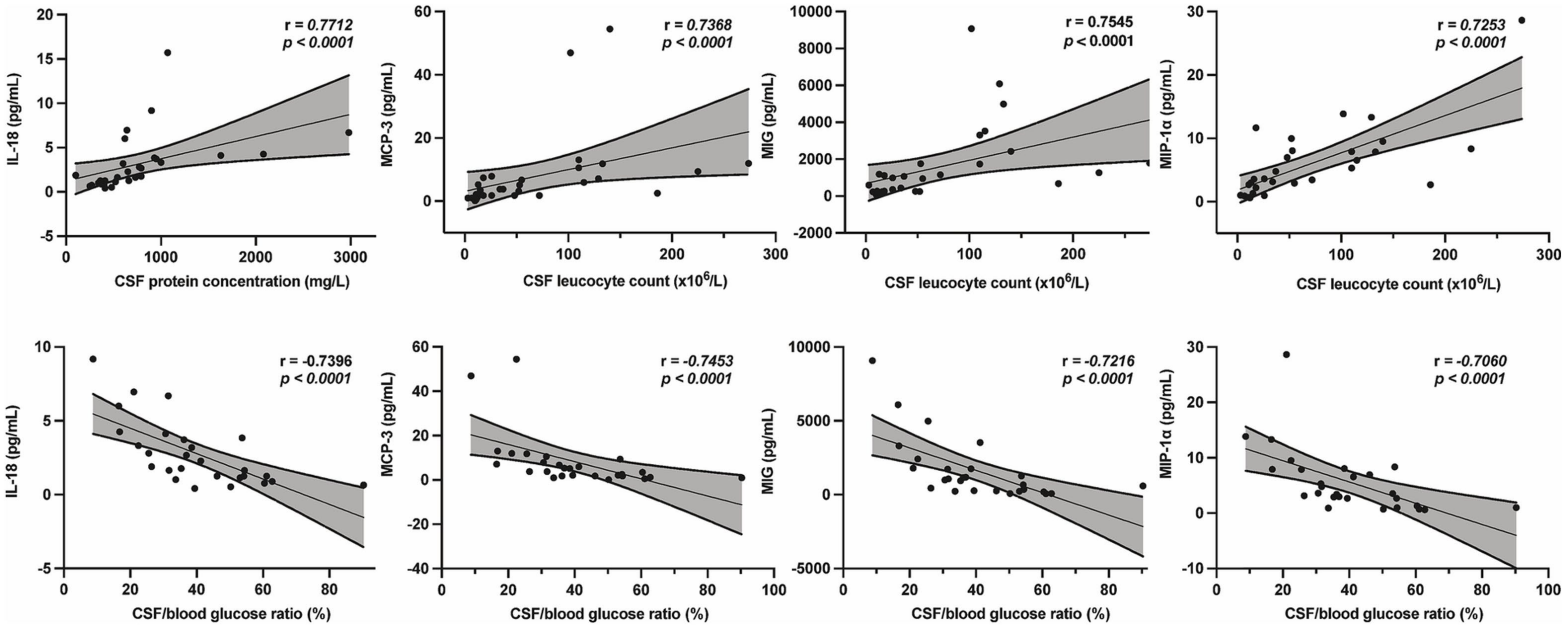
The correlation between baseline CSF parameters and CSF cytokine levels in patients with CM. CSF protein concentration (mg/L) was strongly positively correlated with IL-18, and CSF leucocytes counts (×10^6^/L) were strongly positively correlated with MCP-3, MIG, and MIP-1α. CSF/blood glucose ratio (%) were strongly negatively correlated with MCP-3, MIG, MIP-1, and IL-18. Each dot represents an individual patient sample. Solid lines indicate linear regression fits and shaded areas represent the 95% confidence intervals. Correlation coefficients (*r*) an*d cor*responding *p* values were calculated using Spearman’s correlation analysis. CSF, cerebrospinal fluid; CM, cryptococcal meningitis; IL, interleukin; MCP, monocyte chemotactic protein; MIG, monokine induced by interferon gamma; MIP, macrophage inflammatory protein.

To investigate the relationship between cytokine levels and CSF parameters, we performed a correlation analysis focused on the ten key cytokines. CSF leukocyte count (×10^6^/L) showed moderate positive correlations with IL-4 (*r* = 0.6937, *p* < 0.0001) and IL-18 (*r* = 0.6579, *p* < 0.0001), while IL-17 (*r* = 0.7206, *p* < 0.0001), IL-2r*α* (*r* = 0.8548, *p* < 0.0001), MCP-3 (*r* = 0.7368, *p* < 0.0001), MIG (*r* = 0.7545, *p* < 0.0001), MIP-1α (*r* = 0.7253, *p* < 0.0001), MIP-1β (*r* = 0.7632, *p* < 0.0001), TNF-α (*r* = 0.8828, *p* < 0.0001), and TRAIL (*r* = 0.7875, *p* < 0.0001) exhibited strong positive correlations. Among these, MCP-3 (*r* = −0.7453, *p* < 0.0001), MIG (*r* = −0.7216, *p* < 0.0001), MIP-1α (*r* = −0.7060, *p* < 0.0001), and IL-18 (*r* = −0.7396, *p* < 0.0001) were strongly negatively correlated with the CSF/blood glucose ratio. IL-18 also showed a strong positive correlation with CSF protein concentration (mg/L) (*r* = 0.7712, *p* < 0.0001) (Figure 5). These findings may reflect associations between specific cytokines and both inflammation and disease severity in cryptococcal meningitis.

## 4 Discussion

CM, a life-threatening fungal infection of the CNS, continues to pose a significant global health burden, with high mortality and persistent neurological complications. Notably, the proportion of CM cases occurring in HIV-negative individuals has risen sharply in recent years (24). In East Asia, approximately 65–70% of non-HIV CM cases now occur in patients without identifiable predisposing factors (25). To investigate the immune response to *Cryptococcus* , we conducted CSF cytokine profiling in a well-characterized cohort of HIV-negative CM patients.

We found that CSF cytokine levels were significantly elevated in patients with cryptococcal CNS infection (26). Compared with the NCID group, 39 out of 48 cytokines showed a marked increase in concentration following CNS invasion by *Cryptococcus.* We then assessed cytokine dynamics in 15 patients after antifungal treatment. Compared with the UCM group, 32 cytokines were significantly reduced in the TCM group. Importantly, these same 32 cytokines also showed significant differences between the CM and NCID groups. These findings are consistent with a robust inflammatory response in cryptococcal meningitis and may reflect alterations in the CSF immune environment. In line with prior reports, our findings confirm that patients with CM exhibit significantly higher cytokine levels than those without the disease (27). Specifically, cytokines associated with T helper 1 (Th1; TNF-*α* and IFN-*γ*), Th2 (IL-4, IL-6, and IL-10), and Th17 (IL-17) responses appear to contribute to CM pathogenesis, consistent with evidence of excessive immune activation in CNS infections (28). We applied PCA to explore patterns of correlated cytokines and explain variance within the dataset. In CM patients, PCA revealed correlated immune responses involving Th1, Th2, and Th17 cytokines, with positive loadings for TNF-α, IFN-γ, IL-4, IL-6, IL-10, and IL-17 on PC1 (Supplementary Figure S2). This observation supports prior reports of parallel compensatory increases in Th2-type cytokines during cryptococcal infection (29).

Previous studies have shown that *C. neoformans* infection reduces glucose level in the brain (30). Upon CNS invasion, *C. neoformans* disrupts the blood–brain barrier (BBB), particularly targeting endothelial cell structures, which impairs glucose transport to the brain (31). Additionally, astrocyte-neuron metabolic coupling plays a critical role in brain energy homeostasis (32). The astrocyte proliferation induced by *C. neoformans* may further alter brain glucose metabolism (33). Simultaneously, *C. neoformans* consumes substantial extracellular glucose for proliferation, further depleting glucose availability in the CNS (34). Moreover, glucose in the CSF promotes *C. neoformans* tolerance to amphotericin B by activating the glucose repression regulator MIG1 during cryptococcal meningitis. This finding provides compelling *in vivo* evidence that host-derived metabolites can induce fungicidal tolerance (35). Clinically, a low CSF/blood glucose ratio (%) correlates with poor outcomes including seizures, deep coma, and mortality (36, 37). Interestingly, our analysis identified a significant negative correlation between PC4 and both CSF glucose level and the CSF/blood glucose ratio. Component loading analysis indicated that PC4 is positively influenced by MIP-1α and MIP-1β, suggesting that specific cytokine patterns may be associated with altered CSF glucose dynamics.

Next, we analyzed the correlations between classic CSF parameters and the levels of ten cytokines that previously showed the most significant differences between the TCM and UCM groups. Our results revealed that MIG strongly negatively correlated with the CSF/blood glucose ratio (%) and positively correlated with CSF leukocyte counts in CM patients. MIP-1α and MCP-3 showed similar correlation patterns. IL-18 exhibited a strong positive correlation with CSF protein concentration and a negative correlation with the CSF/blood glucose ratio. Among these, MIG, MIP-1α, MCP-3, and IL-18 emerged as the cytokines most strongly associated with CSF parameters, suggesting potential links to the inflammatory response during cryptococcal infection. These observations provide insight into CSF immune profiles and may help guide future studies investigating immunopathogenic mechanisms in cryptococcal meningitis.

IL-18, originally termed interferon-gamma–inducing factor (38), serves as a crucial bridge between innate and adaptive immunity, regulating both cellular and humoral responses (39–41). IL-18 exerts dual functions in immune regulation: it can drive pathogenic inflammation or promote immune restoration depending on the context (42). Experimental models have shown that IL-18 contributes to host resistance against cryptococcal infection by inducing IFN-*γ* production and enhancing Th1 cell development driven by IL-12, thereby helping to prevent fungal dissemination to the brain (43–46). However, in certain inflammatory settings, IL-18 deficiency has been linked to reduced local inflammation and improved outcomes, emphasizing the necessity for tight regulation of IL-18 activity (47).

In contrast to IL-18, the chemokines MIG (CXCL9), MIP-1*α* (CCL3), and MCP-3 (CCL7) primarily mediate the recruitment and trafficking of immune cells to sites of inflammation (48). MIG, which is induced by IFN-γ (49), attracts monocytes and macrophages and regulates the inflammatory response (50). TNF-α further enhances its expression through a synergistic mechanism (51). By binding to its receptor CXCR3, MIG promotes the recruitment of Th1 lymphocytes to inflamed tissues, thereby playing a central role in the immune response (50). In addition to facilitating Th1 cell migration, MIG downregulates CXCR3 surface expression and induces directional chemotaxis toward inflammatory foci (52). The MIG/CXCR3 signaling axis is essential for T cell differentiation into Th1 subsets and for guiding immune cells to lesion sites, serving as a key regulatory mechanism in immune surveillance (53). This axis also helps immune coordination, as MIG preferentially recruits Th1 cells while inhibiting Th2 cell migration toward CCR3 ligands (54). Furthermore, MIG, in concert with IP-10 (CXCL10) and I-TAC (CXCL11), enhances the local production of TNF-*α* and IFN-*γ* in inflamed tissues, thereby sustaining and amplifying the inflammatory response (55).

MIP-1α (CCL3) and MCP-3 (CCL7), both members of the CC chemokine family, are notably upregulated in CNS infections, suggesting their involvement in neuroinflammatory processes (56, 57). MIP-1α plays a pivotal role in intensifying local immune responses by promoting immune cell infiltration and stimulating cytokine production (58). MIP-1α production reflects both an appropriate immune response and a deficient Th1 response to disseminated *Cryptococcus* infection (59). Elevated MIP-1α levels likely represent a compensatory mechanism by the innate immune system aimed at enhancing fungal clearance. Several studies have linked increased MIP-1α expression to the development of IRIS and a heightened risk of mortality (8). In parallel, experimental studies have shown that MCP-3 is upregulated early during *Cryptococcus i* nfection, where it promotes the recruitment of dendritic cells (DCs) (60). These DCs accumulate in the CNS during inflammation and may actively contribute to local immune responses (61).

Several limitations should be noted in our study. The relatively small sample size in each group, together with the use of one NCID control group comprising heterogeneous non-inflammatory neurological conditions, may limit the generalizability of the findings and contribute to baseline variability in immune profiles. Consequently, the observed cytokine differences should be interpreted cautiously. To address these issues, future studies including larger cohorts and incorporating additional groups with other types of central nervous system infections may help better distinguish CM-specific immune responses and clarify the immunological mechanisms underlying cytokine alterations. Finally, further validation using ELISA and *in vitro* functional assays is necessary to confirm the roles of the identified cytokines and their mechanistic relevance in cryptococcal meningitis.

## 5 Conclusion

Our study identifies distinct CSF cytokine profiles in HIV-negative CM patients compared to those with non-cryptococcal infectious diseases. Among these, MIG, MIP-1α, MCP-3, and IL-18 were most strongly associated with markers of CNS inflammation, suggesting potential links to the host immune response during cryptococcal infection. These observations provide insight into the immunopathogenic mechanisms underlying CM and may inform future studies aimed at developing targeted immunomodulatory strategies.

## Data Availability

The original contributions presented in the study are included in the article/Supplementary material, further inquiries can be directed to the corresponding authors.
