# Supplementary material for: Cerebrospinal fluid cytokine-driven immune responses in HIV-negative cryptococcal meningitis
Source: Front Med (Lausanne). 2026 Apr 7;13:1800744. doi: 10.3389/fmed.2026.1800744 (PMC13095563; doi:10.3389/fmed.2026.1800744)
Supplement: Supplementary file 1 [file Data_Sheet_1.pdf]

Supplementary Material

1 Supplementary Figures

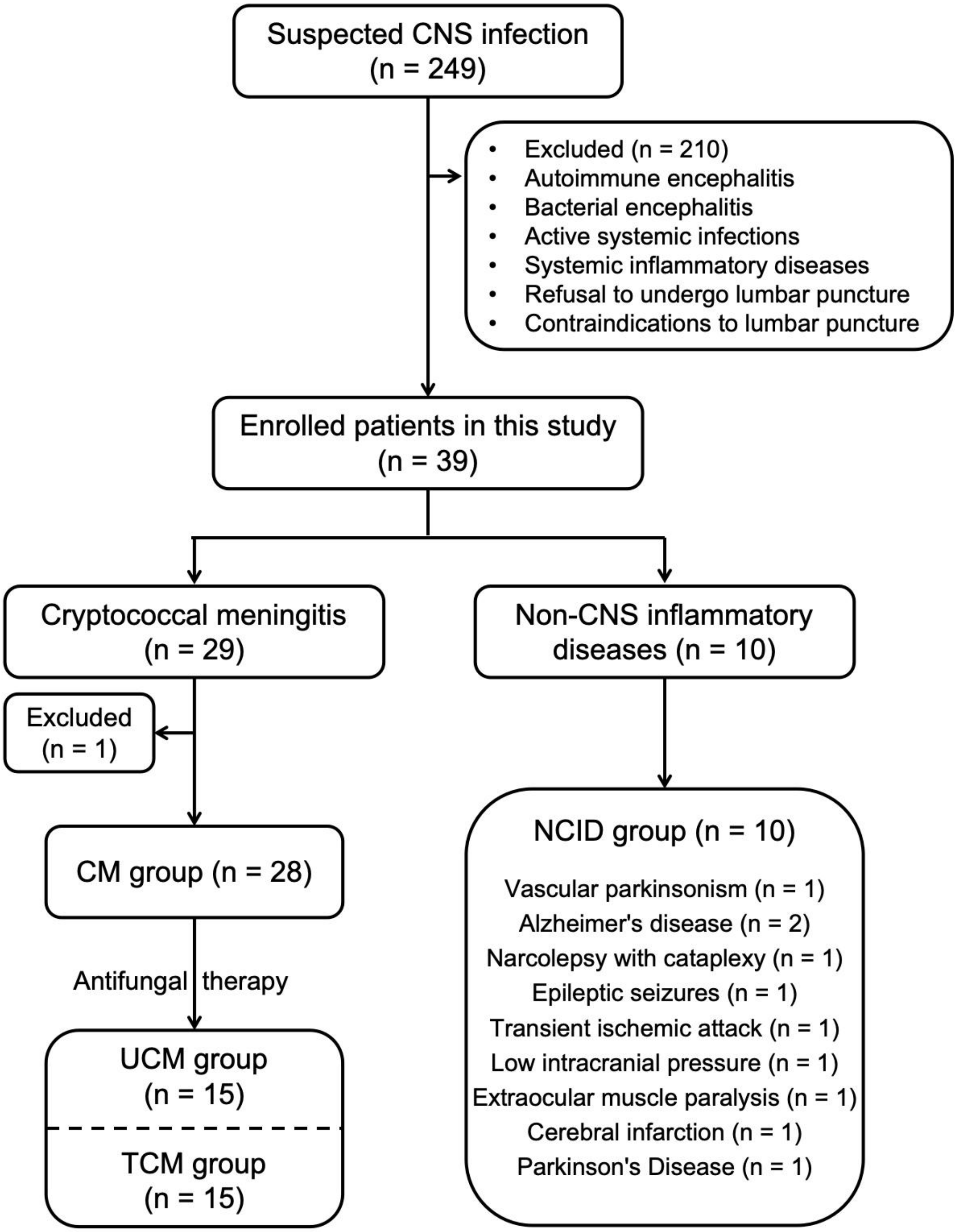

Supplementary Figure 1. Flowchart of the study design and participant classification. Patients with suspected CNS infection were screened for inclusion in this study. Individuals diagnosed with autoimmune encephalitis, bacterial encephalitis, active systemic infections, or systemic inflammatory diseases were excluded, as well as those who refused to undergo lumbar puncture or had contraindications to lumbar puncture. A total of 39 patients were enrolled. Of these patients, 28 were diagnosed with CM, 10 with NCID, and one CM patient was excluded due to an insufficient CSF sample volume for analysis. CSF samples collected after antifungal treatment were available from 15 CM patients. The NCID group included one case of vascular parkinsonism, two cases of Alzheimer's disease, one case of narcolepsy with cataplexy, one case of epileptic seizures, one case of transient ischemic attack, one case of low intracranial pressure, one case of extraocular muscle paralysis, one case of cerebral infarction and one case of Parkinson's disease. CNS, central nervous system; CM, cryptococcal meningitis; NCID, non-CNS inflammatory diseases; UCM, untreated CM; TCM, treated CM; CSF: cerebrospinal fluid.

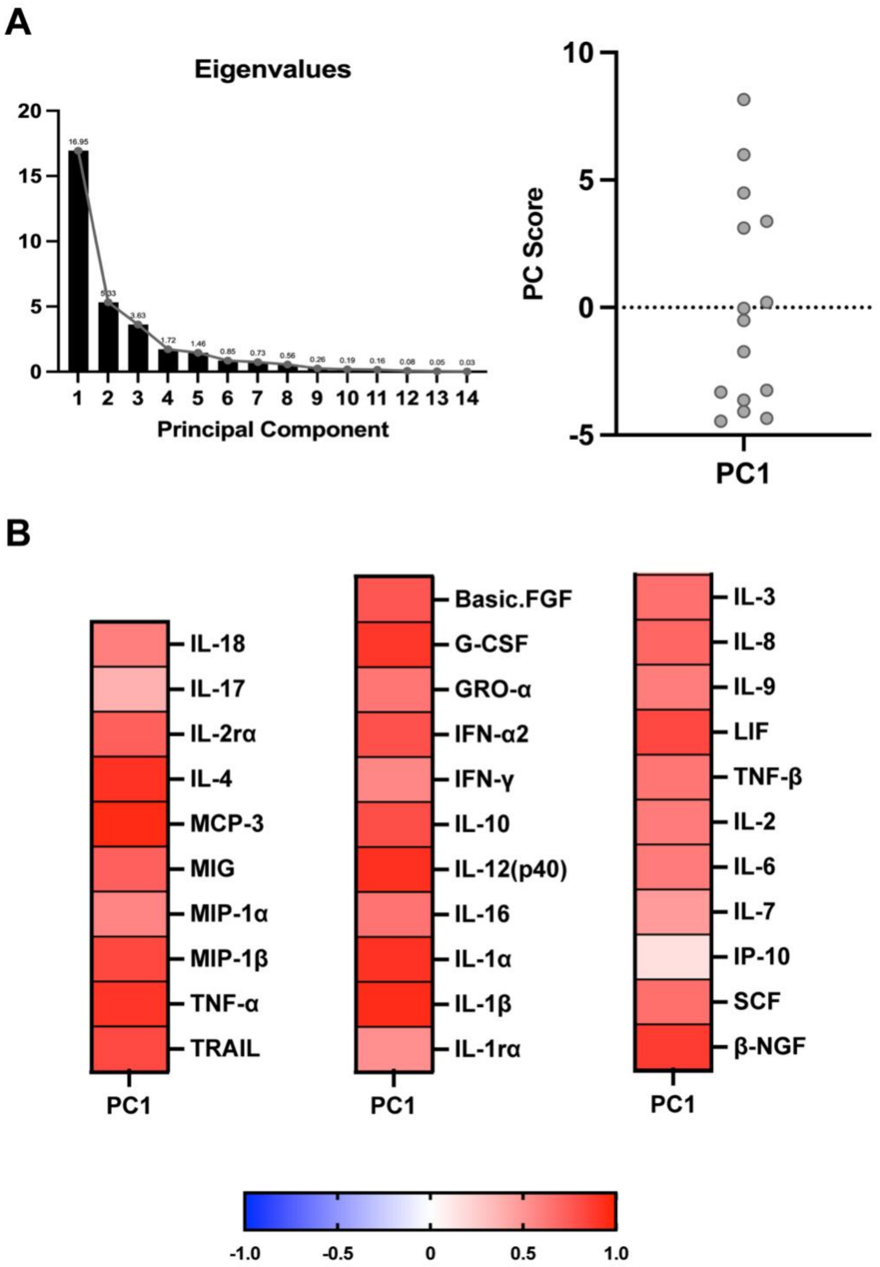

Supplementary Figure 2. Principal component analysis of CSF cytokine profiles in patients with UCM. (A) Proportion of variance explained by each principal component, as well as the distribution of principal component scores among the 15 UCM patients included in the study; (B) The heat map shows the loading values of these cytokines contributing to PC1 in the 15 UCM patients included in the study. Color intensity represents the magnitude and direction of the PC loadings (−1 to 1). Th1, Th2, and Th17 cytokines, including TNF-α, IFN-γ, IL-4, IL-6, IL-10, and IL-17, showed positive loadings on PC1. CSF, cerebrospinal fluid; CM, cryptococcal meningitis; NCID, non-CNS inflammatory diseases; UCM, untreated CM; TCM, treated CM; PC, principal component; Th, T helper; IL, interleukin; MCP, monocyte chemotactic protein; MIG, monokine induced by IFN-γ; MIP, macrophage inflammatory protein; TNF, tumor necrosis factor; TRAIL, TNF-related apoptosis-inducing ligand; FGF, Fibroblast Growth Factor; G-CSF, granulocyte colony-stimulating factor;

GRO, Growth-related oncogene; IFN, interferon; LIF, leukemia inhibitory factor; IP, IFN-γ inducible protein; SCF, stem cell factor; NGF, nerve growth factor.

2 Supplementary Tables

Supplementary Table 1. CSF laboratory parameters of the enrolled patients (n = 38).

No.	P (mmH_2O)	CSF leukocyte ($\times 10^6$/L)	CSF protein (mg/L)	CSF glucose (mmol/L)	CSF/blood glucose ratio (%)
**1**	180	140	1000	1.37	22.46
**2**	>300	153	760	1.11	15.59
**3**	270	91	1180	1.11	21.06
**4**	320	110	2980	1.57	31.46
**5**	70	364	1740	2.06	30.03
**6**	120	26	540	3.96	54.40
**7**	NA	128	3010	0.06	1.21
**8**	150	151	890	1.76	21.13
**9**	>330	129	620	0.97	16.52
**10**	>300	53	600	1.36	38.53
**11**	280	133	770	1.47	25.61
**12**	190	77	1090	1.35	26.52
**13**	178	115	650	2.47	41.24
**14**	160	110	2080	2.16	16.78
**15**	130	55	790	1.76	35.34
**16**	80	225	930	3.92	53.70
**17**	>330	292	800	2.08	30.32
**18**	250	39	640	5.64	39.52
**19**	120	186	410	2.95	54.23
**20**	>330	110	1000	0.02	0.30
**21**	>300	102	900	0.49	8.78
**22**	30	107	1800	4.94	39.43
**23**	210	26	820	5.52	42.46
**24**	218	72	950	2.46	36.18
**25**	120	203	1270	2.96	34.22
**26**	80	274	640	1.15	21.10
**27**	264	52	1070	2.37	NA
**28**	95	171	NA	1.69	NA
**29**	85	2	950	2.71	52.22
**30**	70	1	330	3.27	NA
**31**	120	1	480	3.56	72.36
**32**	130	1	190	3.37	66.47
**33**	70	1	340	3.33	72.55
**34**	80	3	320	3.06	67.85
**35**	120	1	290	4.05	63.98
**36**	80	1	190	5.64	89.81
**37**	140	0	370	3.49	56.02
**38**	162	1	310	3.19	49.30

No., case number; P, Intracranial pressure; CSF, cerebrospinal fluid; NA, not available.

Supplementary Table 2. Changes in CSF laboratory parameters in 15 CM patients before and after antifungal therapy.

**No.**	**UCM**					**TCM**				
	**P (mmH_2O)**	**CSF leukocyte ($\times10^6$/L)**	**CSF glucose (mmol/L)**	**CSF protein (mg/L)**	**CSF/blood glucose ratio (%)**	**P (mmH_2O)**	**CSF leukocyte ($\times10^6$/L)**	**CSF glucose (mmol/L)**	**CSF protein (mg/L)**	**CSF/blood glucose ratio (%)**
**1**	180	140	1.4	1000	22.46	160	34	1.8	<100	26.39
**4**	320	110	1.6	2980	31.46	330	26	1.9	1630	30.66
**6**	120	26	4.0	540	54.40	130	3	3.7	250	90.31
**9**	>330	129	1.0	620	16.52	170	16	2.8	520	53.10
**10**	>300	53	1.4	600	38.53	260	37	2.7	730	31.68
**11**	280	133	1.5	770	25.61	230	10	2.9	480	50.26
**13**	178	115	2.5	650	41.24	130	7	3.1	340	33.69
**14**	160	110	2.2	2080	16.78	95	13	2.3	790	36.91
**15**	130	55	1.8	790	35.34	155	11	3.6	360	61.11
**16**	80	225	3.9	930	53.70	210	15	3.4	270	60.43
**19**	120	186	3.0	410	54.23	190	18	3.2	400	NA
**21**	>330	102	0.5	900	8.78	175	11	2.8	410	39.44
**24**	218	72	2.5	950	36.18	190	12	3.2	370	62.77
**26**	80	274	1.2	640	21.10	130	48	3.6	660	46.12
**27**	264	52	2.4	1070	NA	310	18	NA	NA	NA

UCM, untreated cryptococcal meningitis; TCM, treated cryptococcal meningitis; No., case number; P, Intracranial pressure; CSF, cerebrospinal fluid; NA, not available.

Supplementary Table 3. Concentration of cytokines (pg/mL) in CSF between the CM and the NICD groups and the UCM and the TCM groups.

Cytokines	CM (n = 28)	NICD (n = 10)	UCM (n = 15)	TCM (n = 15)	CM vs. NCID *p* value	UCM vs. TCM *p* value
**Basic FGF**	5.52 (4.73, 6.66)	1.79 (1.79, 1.79)	5.52 (2.73, 5.79)	1.79 (1.33, 2.73)	< 0.0001	0.0056
**CTACK**	17.61 (14.09, 21.12)	6.15 (3.48, 8.36)	15.85 (14.09, 18.49)	12.33 (11.45, 18.49)	< 0.0001	0.1388
**Eotaxin**	8.84 (6.64, 9.86)	3.43 (2.69, 5.41)	8.73 (6.17, 9.50)	8.21 (6.67, 11.26)	0.0009	0.6257
**G-CSF**	67.47 (49.23, 94.93)	6.25 (4.86,7.54)	55.10 (41.37, 106.23)	26.09 (12.21, 33.23)	< 0.0001	0.0015
**GM-CSF**	0.15 (0.15, 0.41)	0.15 (0.15, 0.15)	0.15 (0.15, 0.23)	0.15 (0.15, 0.15)	0.1773	0.9219
**GRO-α**	544.64 (259.95, 328.06)	26.28 (26.28, 26.28)	533.49 (346.77, 1143.73)	37.43 (26.28, 240.60)	< 0.0001	0.0040
**HGF**	240.04 (202.20, 1356.98)	184.24 (161.11, 268.70)	202.78 (184.23, 244.72)	207.42 (153.05, 297.46)	0.0690	0.9508
**IFN-α2**	2.07 (1.70, 2.33)	0.69 (0.69, 0.69)	1.70 (0.80, 2.17)	0.69 (0.69, 1.49)	< 0.0001	0.0033
**IFN-γ**	73.26 (34.94, 104.86)	0.81 (0.36, 1.08)	42.70 (18.28, 73.69)	7.38 (3.42, 13.61)	< 0.0001	0.0022
**IL-10**	47.65 (23.21, 63.65)	0.63 (0.18, 0.88)	25.93 (15.19, 41.35)	3.80 (0.88, 10.12)	< 0.0001	0.0020
**IL-12 (p40)**	16.70 (10.68, 21.23)	2.65 (1.15, 4.66)	14.69 (9.17, 17.71)	6.66 (4.66, 12.19)	< 0.0001	0.0049
**IL-12 (p70)**	0.78 (0.35, 0.91)	0.91 (0.91, 0.91)	0.45 (0.21, 0.91)	0.54 (0.16, 0.91)	0.2826	0.8795
**IL-13**	0.91 (0.65, 1.28)	1.51 (1.38, 1.78)	1.07 (0.64, 1.56)	0.99 (0.58, 1.88)	0.0207	0.4228
**IL-15**	11.40 (11.40, 11.40)	11.40 (11.40, 11.40)	11.40 (11.40, 11.40)	11.40 (11.40, 11.40)	0.5411	0.7500
**IL-16**	24.21 (17.88, 42.72)	2.34 (1.23, 2.70)	23.42 (14.87, 42.74)	8.47 (4.89, 14.94)	< 0.0001	0.0034
**IL-17**	10.08 (6.05, 16.54)	1.52 (1.52, 1.52)	9.68 (4.62, 15.69)	1.75 (1.44, 4.02)	< 0.0001	0.0004
**IL-18**	4.22 (3.29, 7.26)	0.51 (0.34, 0.64)	3.72 (2.54, 6.35)	1.14 (0.77, 1.77)	< 0.0001	< 0.0001
**IL-1α**	17.38 (13.18, 21.87)	1.54 (0.60, 1.54)	15.86 (7.26, 20.39)	3.95 (1.54, 10.45)	< 0.0001	0.0011
**IL-1β**	2.26 (1.98, 2.66)	0.41 (0.29, 0.41)	2.10 (1.21, 2.50)	0.89 (0.57, 1.57)	< 0.0001	0.0027
**IL-1rα**	2212.70 (1097.90, 3729.68)	16.94 (14.90, 24.50)	1760.96 (439.56, 2422.38)	83.48 (48.73, 446.34)	< 0.0001	0.0026
**IL-2**	3.03 (2.03, 3.99)	1.31 (0.61, 1.31)	2.06 (1.22, 3.72)	1.31 (0.66, 1.57)	< 0.0001	0.021
**IL-2rα**	49.70 (30.47, 78.77)	0.56 (0.34, 0.94)	37.76 (19.08, 71.65)	6.93 (2.72, 17.54)	< 0.0001	0.0008
**IL-3**	0.63 (0.44, 0.88)	0.02 (0.02, 0.05)	0.53 (0.32, 0.85)	0.27 (0.12, 0.40)	< 0.0001	0.0022
**IL-4**	2.66 (2.45, 3.26)	0.66 (0.55, 0.66)	2.52 (1.75, 2.95)	1.24 (0.87, 1.99)	< 0.0001	0.0004
**IL-5**	4.42 (4.42, 28.07)	4.42 (4.42, 4.42)	4.42 (4.42, 20.88)	4.42 (4.42, 4.42)	0.0308	0.1563
**IL-6**	152.74 (31.96, 234.75)	0.56 (0.27, 1.85)	68.75 (16.48, 190.18)	4.20 (2.27, 15.34)	< 0.0001	0.0256
**IL-7**	20.35 (11.10, 23.99)	3.88 (3.88, 3.88)	17.41 (11.10, 23.28)	11.10 (7.49, 14.26)	< 0.0001	0.0231
**IL-8**	356.16 (213.42, 702.49)	17.56 (13.13, 22.65)	241.36 (128.68, 586.93)	108.04 (49.26, 157.80)	< 0.0001	0.0015
**IL-9**	36.09 (18.88, 44.81)	6.42 (3.77, 8.97)	29.44 (28.88, 48.31)	13.86 (11.44, 27.70)	< 0.0001	0.0038
**IP-10**	44235.61 (37091.91, 87810.57)	1411.00 (1216.83, 1843.92)	54273.56 (40284.54, 100766.34)	19991.69 (8503.31, 25151.14)	< 0.0001	0.0222
**LIF**	43.78 (29.48, 47.96)	20.4 (1.36, 2.71)	35.28 (23.55, 47.68)	11.08 (6.35, 26.53)	< 0.0001	0.0013
**M-CSF**	14.91 (8.09, 17.86)	6.29 (4.97, 7.51)	13.32 (6.50, 15.91)	8.67 (6.66, 11.63)	< 0.0001	0.3024
**MCP-1**	113.67 (85.33, 246.13)	97.17 (66.94, 110.99)	89.03 (67.22, 121.61)	138.61 (110.32, 174.11)	0.2054	0.0833
**MCP-3**	11.55 (7.04, 19.87)	0.52 (0.12, 0.87)	7.14 (4.14, 11.89)	2.15 (1.10, 3.79)	< 0.0001	0.0009
**MIF**	274.32 (201.69, 500.60)	306.17 (223.08, 353.32)	341.44 (208.96, 481.65)	344.38 (234.36, 416.24)	0.7455	0.8722
**MIG**	2986.02 (1750.11, 4478.55)	44.52 (35.82, 73.73)	1755.01 (1050.74, 3417.80)	266.33 (208.33, 792.75)	< 0.0001	0.0009
**MIP-1α**	9.88 (7.82, 13.46)	0.33 (0.19, 0.66)	7.92 (4.37, 9.74)	2.69 (0.96, 3.59)	< 0.0001	0.0005
**MIP-1β**	50.15 (33.84, 60.59)	5.53 (4.69, 6.74)	37.91 (32.39, 55.80)	19.52 (10.42, 28.81)	< 0.0001	0.0006
**PDGF-BB**	38.62 (35.45, 45.87)	18.77 (11.75, 25.43)	35.45 (24.67, 37.82)	32.32 (17.36, 38.62)	< 0.0001	0.4558
**RANTES**	12.82 (6.91, 22.28)	0.79 (0.79, 0.79)	13.34 (8.72, 16.82)	3.17 (0.79, 14.93)	0.0002	0.4263
**SCF**	16.22 (12.39, 22.13)	9.99 (4.99, 12.10)	14.00 (9.92, 20.59)	11.28 (7.99, 14.93)	0.0010	0.0157
**SCGF-β**	13647.15 (9803.82, 18144.32)	14830.31 (10312.25, 18215.68)	9910.36 (8,891.98, 13742.09)	10289.04 (8157.02, 13032.39)	0.8259	0.2552

**SDF-1α**	1460.54 (1265.61, 1568.43)	1195.42 (1103.88, 1339.14)	1484.16 (1381.01, 1562.53)	1408.23 (1171.58, 1679.77)	0.0710	0.9150
**TNF-α**	245.62 (167.78, 375.91)	5.02 (1.23, 8.69)	197.83 (124.39, 365.24)	42.37 (17.54, 67.06)	< 0.0001	0.0004
**TNF-β**	36.64 (29.36, 45.47)	5.05 (4.37, 5.85)	32.76 (29.12, 45.11)	14.09 (8.49, 26.91)	< 0.0001	0.0027
**TRAIL**	3.50 (2.91, 4.86)	0.93 (0.45, 0.93)	3.07 (2.25, 4.50)	1.45 (0.93, 1.98)	< 0.0001	0.0006
**VEGF**	4.12 (4.12, 10.09)	4.12 (4.12, 4.12)	4.12 (4.12, 6.18)	4.12 (4.12, 4.12)	0.0947	0.3125
**β-NGF**	3.02 (1.93, 3.55)	0.14 (0.09, 0.36)	2.31 (1.72, 3.26)	1.84 (0.43,2.20)	< 0.0001	0.0144

Data were presented as median (interquartile range). Two groups were compared using Student's t-test or nonparametric tests, as appropriate. P values were adjusted for multiple comparisons using the Benjamini–Hochberg false discovery rate (FDR) correction. CSF, cerebrospinal fluid; CM, cryptococcal meningitis; NCID, non-CNS inflammatory diseases; UCM, untreated CM; TCM, treated CM; Basic FGF, Basic Fibroblast Growth Factor; CTACK, cutaneous T cell-attracting chemokine; Eotaxin, eosinophil chemotactic protein; G-CSF, granulocyte colony-stimulating factor; GM-CSF, granulocyte-macrophage colony-stimulating factor; GRO, Growth-related oncogene; HGF, Hepatocyte growth factor; IFN, interferon; IL, interleukin; IP, IFN-γ inducible protein; LIF, leukemia inhibitory factor; M-CSF, macrophage-colony stimulating factor; MCP, monocyte chemotactic protein; MIF, macrophage migration inhibitory factor; MIG, monokine induced by IFN-γ; MIP, macrophage inflammatory protein; PDGF, platelet-derived growth factor; RANTES, regulated upon activation normal T cell expressed and secreted factor; SCF, stem cell factor; SCGF, stem cell growth factor; SDF, stromal cell derived factor; TNF, tumor necrosis factor; TRAIL, TNF-related apoptosis-inducing ligand; VEGF, vascular endothelial growth factor; NGF, nerve growth factor.
